# Depth drives the distribution of microbial ecological functions in the coastal western Antarctic Peninsula

**DOI:** 10.3389/fmicb.2023.1168507

**Published:** 2023-05-18

**Authors:** Avishek Dutta, Elizabeth Connors, Rebecca Trinh, Natalia Erazo, Srishti Dasarathy, Hugh W. Ducklow, Deborah K. Steinberg, Oscar M. Schofield, Jeff S. Bowman

**Affiliations:** ^1^Integrative Oceanography Division, Scripps Institution of Oceanography, University of California, San Diego, La Jolla, CA, United States; ^2^Department of Geology, University of Georgia, Athens, GA, United States; ^3^Savannah River Ecology Laboratory, University of Georgia, Aiken, SC, United States; ^4^Department of Earth and Environmental Sciences, Lamont-Doherty Earth Observatory, Columbia University, Palisades, NY, United States; ^5^Department of Biological Science, College of William & Mary, Virginia Institute of Marine Science, Gloucester Point, VA, United States; ^6^Department of Marine and Coastal Sciences, Rutgers University, New Brunswick, NJ, United States; ^7^Center for Microbiome Innovation, University of California, San Diego, La Jolla, CA, United States

**Keywords:** metagenomics, Antarctic microbiome, Palmer LTER, metagenome-assembled genomes (MAGs), microbial community function

## Abstract

The Antarctic marine environment is a dynamic ecosystem where microorganisms play an important role in key biogeochemical cycles. Despite the role that microbes play in this ecosystem, little is known about the genetic and metabolic diversity of Antarctic marine microbes. In this study we leveraged DNA samples collected by the Palmer Long Term Ecological Research (LTER) project to sequence shotgun metagenomes of 48 key samples collected across the marine ecosystem of the western Antarctic Peninsula (wAP). We developed an *in silico* metagenomics pipeline (iMAGine) for processing metagenomic data and constructing metagenome-assembled genomes (MAGs), identifying a diverse genomic repertoire related to the carbon, sulfur, and nitrogen cycles. A novel analytical approach based on gene coverage was used to understand the differences in microbial community functions across depth and region. Our results showed that microbial community functions were partitioned based on depth. Bacterial members harbored diverse genes for carbohydrate transformation, indicating the availability of processes to convert complex carbons into simpler bioavailable forms. We generated 137 dereplicated MAGs giving us a new perspective on the role of prokaryotes in the coastal wAP. In particular, the presence of mixotrophic prokaryotes capable of autotrophic and heterotrophic lifestyles indicated a metabolically flexible community, which we hypothesize enables survival under rapidly changing conditions. Overall, the study identified key microbial community functions and created a valuable sequence library collection for future Antarctic genomics research.

## 1. Introduction

Marine microorganisms play an important role in regulating biogeochemical cycles (Green et al., 2008). They are key drivers of the transformation of carbon-, nitrogen-, and sulfur-containing compounds in the environment. Changes in environmental conditions impact microbial communities, which in turn exert control over many environmental parameters (Thompson et al., 2017; Dutta et al., 2022). The ecological outcomes of the rapid environmental change are well documented for the western Antarctic Peninsula (wAP) (Meredith and King, 2005; Clarke et al., 2007; Bowman et al., 2016, 2017), home to multiple long-term observing programs. Large shifts in bacterial production relative to primary production signal radically different outcomes for primary production from one year to another (Bowman et al., 2016). The metabolic potential of the heterotrophic bacterial community is presumed to play a strong role in determining what primary production gets recycled by the microbial food web. However, we know little about the genomic makeup of the bacteria and archaea responsible for bacterial production and other marine microbial processes along the wAP (Bowman et al., 2018).

Heterotrophic bacterial populations are intimately linked to phytoplankton blooms and play an essential role in the transformation of phytoplankton-derived organic matter (Buchan et al., 2014). Phytoplankton are a direct source of dissolved organic carbon (DOC) for heterotrophic bacteria in the photic zone. Below the photic zone, heterotrophs reprocess DOC and degrade sinking particles to generate new DOC. The timing of the seasonal phytoplankton bloom, its composition, and its intensity are strongly influenced by physical processes along the wAP. For example, conditions that favor large diatoms are thought to transfer carbon more efficiently to krill and upper trophic-level consumers (Saba et al., 2014). Alternatively, strong winds and reduced sea ice cover can lead to lower levels of primary production and smaller phytoplankton cells, in turn leading to high rates of bacterial production compared to primary production (Bowman et al., 2016). Recent trends and future climate scenarios suggest an increase in wind and a reduction in sea ice for the Antarctic Peninsula (Siegert et al., 2019) and, presumably, a strengthened microbial food web. To better understand the metabolic capabilities of wAP marine bacterial communities, we applied metagenomics to a historic sample library of microbial DNA collected by the Palmer Long Term Ecological Research (LTER) project to better understand how bacterial communities will respond to future environmental change along the wAP.

Most primary production along the wAP is attributed to eukaryotic phytoplankton (Schofield et al., 2018; Lin et al., 2021). However, dark carbon fixation is likely to be a significant process below the photic zone and during the polar night. Though well appreciated for the global ocean (Baltar and Herndl, 2019), surprisingly little is known about the distribution of prokaryotic carbon fixation mechanisms in the Antarctic marine environment. Previous analysis of fosmid libraries from contrasting summer and winter communities along the wAP identified gammaproteobacterial sulfur-oxidizing (GSO) chemolithotrophs (Grzymski et al., 2012). Other works using 16S rRNA gene surveys have shown these taxa to be widely distributed in the coastal Antarctic (Bowman et al., 2017; Bowman and Deming, 2017). Alternate prokaryotic carbon fixation strategies for the wAP may rely on energy obtained from nitrification (Bowman et al., 2016). This study aimed to understand the microbial community functions in the marine ecosystem of coastal wAP. We applied a novel analytical approach based on gene coverage to investigate the distribution of genes and reconstructed metagenome-assembled genomes (MAGs) to understand the pathways associated with prokaryotic carbon fixation and utilization. We combined observations of genes diagnostic of carbon fixation with genes for catabolic processes to identify autotrophic, mixotrophic, and heterotrophic guilds among wAP marine prokaryotes.

## 2. Methods

### 2.1. Sample collection

**Figure 1:**
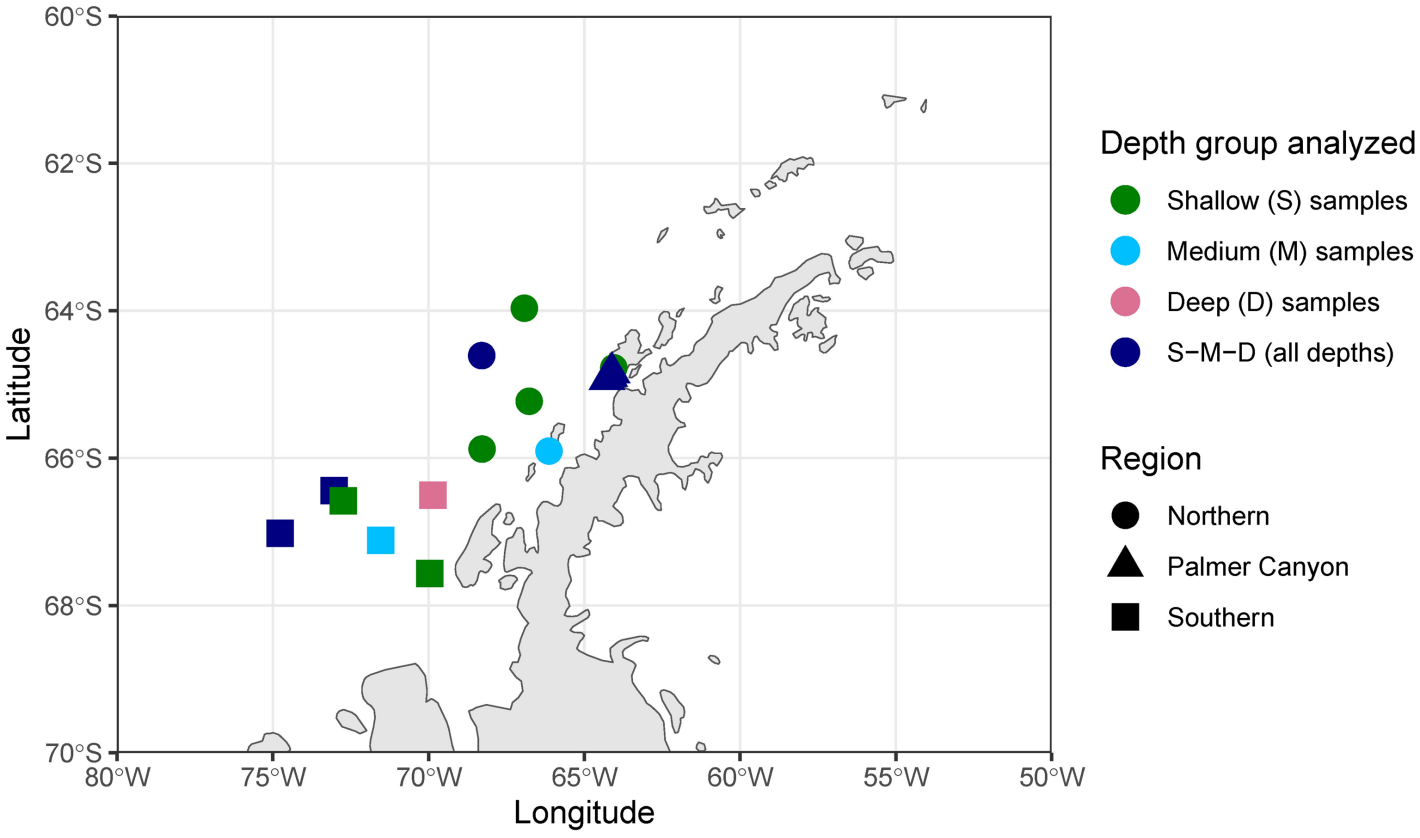
Sample locations in the western Antarctic Peninsula. S, only shallower samples analyzed; M, only medium-depth samples analyzed; D, only deeper samples analyzed; S-M-D, samples collected from deep, medium, and shallow environments analyzed.

Forty-eight samples were selected covering different depth horizons (0–275 m) on the Palmer LTER sampling grid (lines 100–600, covering latitudes 67.566533°S to 63.96565°S) (Waters and Smith, 1992) and locations of special significance such as Palmer Canyon, Armstrong Reef, and the coastal LTER time-series near Palmer Station (Figure 1). The selection of samples was made based on three different parameters: (i) higher abundances of unclassified taxa based on 16S rRNA amplicon sequence data (16S rRNA gene sequence data submitted under NCBI BioProject ID PRJNA901488), (ii) depth profile, and (iii) variations in latitude. A detailed description of the samples used in this study is given in Supplementary Table S1. Forty-two samples were collected during the austral summer of 2019–2020, and four samples were collected during the austral summer of 2018–2019. The remaining two samples were collected in January 2017 and November 2018. We grouped the samples into three depth horizons: shallow (0–40 m), medium (50–75 m), and deep (100–275 m). The depth categorization was done based on mixed layer depth, temperature, and salinity, as reported previously in Schofield et al. (2018) and Seyitmuhammedov et al. (2022). There were 23 samples collected from the shallower depth, whereas 11 and 14 samples were collected from the medium-depth and deeper horizons, respectively. The samples were also categorized into three regions based on Palmer LTER lines: Northern (Line 400 and north of Line 400), Southern (south of Line 400), and Palmer Canyon (near Palmer Station on Anvers Island) (Supplementary Table S1). There were 14 samples collected from the Northern region, whereas 16 and 18 samples were collected from Palmer Canyon and the Southern region, respectively. For each sample, approximately 1 L of seawater was filtered through a sterile 0.2 μm Supor membrane disc filter (Pall Corporation, Port Washington, NY, United States) and stored at −80°C until extraction.

### 2.2. DNA extraction, sequencing, and metagenome analysis

DNA was extracted from the filters using the MagMAX Microbiome Ultra nucleic acid extraction kit and KingFisher Flex extraction system following the manufacturer’s protocols. The extracted DNA was sequenced at the UC San Diego Microbiome Core for shotgun metagenome sequencing on the Illumina NovaSeq platform. Sequencing was done across multiple lanes in two runs (24 samples for each run). The average depth of sequencing for the second run (an average of ~427 million paired-end reads per sample) was higher compared to the first run (an average of ~55 million paired-end reads per sample) to facilitate a separate analysis that will be reported elsewhere. To avoid memory limitation, samples from the second run were down-sampled using reformat.sh, a script from the BBMap package (Bushnell, 2014), to ~55 million paired-end reads per sample. The raw metagenomic data for 48 samples were processed and analyzed using the *in silico* Metagenomics Pipeline (iMAGine) using default parameters.^1^ iMAGine uses fastp (Chen et al., 2018) for filtering, metaSPAdes (Nurk et al., 2017) for assembling the reads, QUAST (Gurevich et al., 2013) for analyzing the assembly quality, bwa-mem (v0.7.17) for aligning the raw reads to the assembly (Li, 2013), samtools for modifying alignment files (Li et al., 2009), metabat2 (Kang et al., 2019) for binning contigs, and checkM (Parks et al., 2015) for quality assessment of the bins. The assembled contigs from iMAGine were used for further analyses.

Genes were predicted from contigs with Prodigal v2.6.3 (Hyatt et al., 2010) using the “meta” flag. The predicted genes were annotated using emapper v2.1.5 (Cantalapiedra et al., 2021) based on a Diamond search (Buchfink et al., 2014). The following arguments were used for search filtering in emapper: --evalue 0.001 (e-value threshold), --score 60 (minimum hit bit score), --pident 40 (minimum percentage identity), --query_cover 20 (minimum percentage query coverage), and --subject_cover 20 (minimum percentage subject coverage). The database used for annotation was eggNOG DB v5.0.2 (Huerta-Cepas et al., 2019). For this study, taxfin.sh (a part of iMAGine) was used for filtering out genes not affiliated to domain bacteria and archaea. Coverage of each gene (average gene fold) was determined using gene_fold_counter.sh (a part of iMAGine), which takes in the alignment map file (sam output from iMAGine), removes unmapped reads and reads mapped to multiple locations using samtools (with parameters -F 0 × 904), uses pileup.sh script from the BBMap package to calculate the average coverage of the contigs and maps back average contig coverage to the genes on those contigs. To enable comparison across metagenomes, all the genes were scaled based on Equation (1):


(1) $$\begin{array}{c} Normalizedgene\\ coverageof\;a\;sample \end{array}=\frac{\;Totalaveragegenecoverage}{\;TotalrpoB\;\left(K03043\right)\;coverage\;}\;$$

For gene-specific analysis, KEGG orthologs from the emapper outputs were considered. Genes mapping to more than one ortholog were not considered for the analysis. Key genes involved in different processes of carbon, nitrogen, and sulfur cycles were selected based on KEGG pathways, whereas genes involved in carbohydrate transformation were selected based on previously published literature (Bergauer et al., 2018). Detailed gene inventories, along with the associated pathways considered in this study, are mentioned in Supplementary Table S2 and Supplementary Figures S1–S4. The KEGG ortholog for *rpoB*, i.e., K03043 was used as a reference for normalization in this study. The genes involved in methane oxidation considered in this study (*pmoA-amoA*, *pmoB-amoB*, and *pmoC-amoC*) also play an important role in ammonia oxidation (a key step in nitrification). Similarly, it has been seen before that the variations of the same gene responsible for dissimilatory sulfate reduction (considered in this study) are also involved in sulfur oxidation (Loy et al., 2009).

Bins with completeness higher than 70% and contamination lower than 5% were considered MAGs. Similar cutoffs were used in a previous study (Parks et al., 2017). For further analysis, MAGs were dereplicated with dRep v3.2.2 (Olm et al., 2017) using a secondary ANI threshold of 0.96 and goANI as the algorithm for secondary clustering. The minimum genome completeness was set to 70%, and the maximum genome contamination was set to 5% for dereplication. All the other dereplication parameters were kept default. The taxonomy of the dereplicated MAGs was assessed using the GTDB-Tk v1.5.0 based on reference database version release 202 (Chaumeil et al., 2020). The multiple sequence alignment output of domain-specific marker genes from GTDB-Tk was used for constructing a phylogenomic tree with RAxML-ng (Kozlov et al., 2019). Tree construction used the WAG amino-acid substitution model and MAGs belonging to Chloroflexota and Thermoproteota as outgroups for bacteria- and archaea-specific phylogenomic trees, respectively. The default number of starting trees was used (10 random and 10 parsimony-based) and 1,000 bootstrap replicates were used for confidence scoring of the final tree.

The number of reads mapped to each MAG was calculated using the mag_abund.py script present in the iMAGine repository,^2^ and the average reads per secondary cluster (as obtained from dRep analyses) was obtained to understand average reads mapped per dereplicated MAGs.

Sequence data for metagenomics reads from 48 samples, along with 137 dereplicated MAGs, can be found under NCBI BioProject ID PRJNA894514. The MAGs have been deposited at GenBank under the accession JAPKAB000000000- JAPKFH000000000.

### 2.3. Determination of putative metabolic lifestyle in MAGs

Functional annotation of MAGs was conducted using two different methods. To identify functional guilds, genes were predicted using Prodigal v2.6.3 and annotated using Ghost Koala (Kanehisa et al., 2016), whereas DRAM 1.4.0 was used to assess pathway completeness and categorize different microbial metabolisms (Shaffer et al., 2020). Though it is hard to ascertain the exact metabolic lifestyle of the MAGs given the limitation imposed by genome completeness, we described the putative metabolisms based on combinations of diagnostic metabolic pathways. All the annotations for metabolic lifestyle assessment were based on DRAM analysis. Glucose utilization (GU) and carbon fixation (CF) pathways having greater than 70% completeness in a MAG were considered in this study. GU, indicative of the use of exogenous fixed carbon, was assessed by the presence of the Embden-Meyerhof pathway and/or the Entner-Doudoroff pathway. CF was assessed based on the presence of either of the three carbon fixing pathways (3-Hydroxypropionate bicycle, Arnon–Buchanan cycle, and Calvin cycle) found in the MAGs. Sulfur oxidation (SO) capability was determined based on the presence of the SOX system and/or the presence of the *dsr* gene. Nitrification (NI) capability was determined based on the presence of either ammonia oxidation genomic repertoire and/or gene involved in the conversion of nitrite to nitrate. Denitrification (DNR) capability was determined based on the presence of either one of the genes involved in the following processes: (i) genes involved in the conversion of nitrate to nitrite, (ii) genes involved in the conversion of nitrite to nitric oxide, and/or (iii) genes involved in the conversion of nitric oxide to nitrous oxide. We described each MAG as a putative autotroph, mixotroph, or heterotroph based on the presence of diagnostic metabolic pathways. Autotrophs were determined by the presence of CF and absence of GU, heterotrophs by the presence of GU and absence of CF, and mixotrophs by the presence of both GU and CF.

### 2.4. Statistical analyses

All the statistical analyses were carried out in R and R Studio (RStudio Team, 2015). Principal Coordinates Analysis (PCoA) of Bray-Curtis dissimilatory based on the normalized gene coverage across different samples was performed using the phyloseq package (McMurdie and Holmes, 2013). For this PCoA, we considered key genes responsible for carbon, nitrogen, sulfur, and carbohydrate transformations (Supplementary Figures S1–S4). A PERMANOVA test was conducted using the “adonis2” function of the Vegan package (Oksanen et al., 2007) to test whether different sample groups had different centroids. The average distance from the median was calculated based on a dispersion test using the “betadisper” function of the Vegan package, which was followed by a permutation test of multivariate homogeneity of group dispersions using the “permutest” function of the Vegan package. Boxplots were made to analyze the differences in process abundances across different depth profiles. The Kruskal-Wallis test for significance was used to determine whether the overall changes were significant, whereas the Wilcoxon test was used to find the pairwise significance. Heatmap and cluster analyses were carried out with the pheatmap package (Kolde, 2019) and based on normalized gene coverage. Column clustering, which displayed the clustering of different samples, was based on Bray–Curtis dissimilatory. Each row, depicting normalized gene coverage, was scaled using min-max scaling and clustered based on correlation. To understand the clustering of MAGs based on genomic repertoire, genes obtained from Ghost Koala analysis were mapped to each dereplicated MAGs using custom R scripts, and Non-Metric Multidimensional Scaling (NMDS) of a binary matrix based on the presence or absence of all genes constraining different metabolic categories were performed using Vegan. All the statistical analyses for the MAGs were conducted based on dereplicated MAGs, except for the mapped read coverage analysis. For mapped read coverage, all the MAGs were considered. A web-based tool^3^ was used to generate Venn diagrams to find the overlap across different metabolic categories to determine the metabolic lifestyle of the MAGs.

## 3. Results

### 3.1. Microbial community function

**Figure 2:**
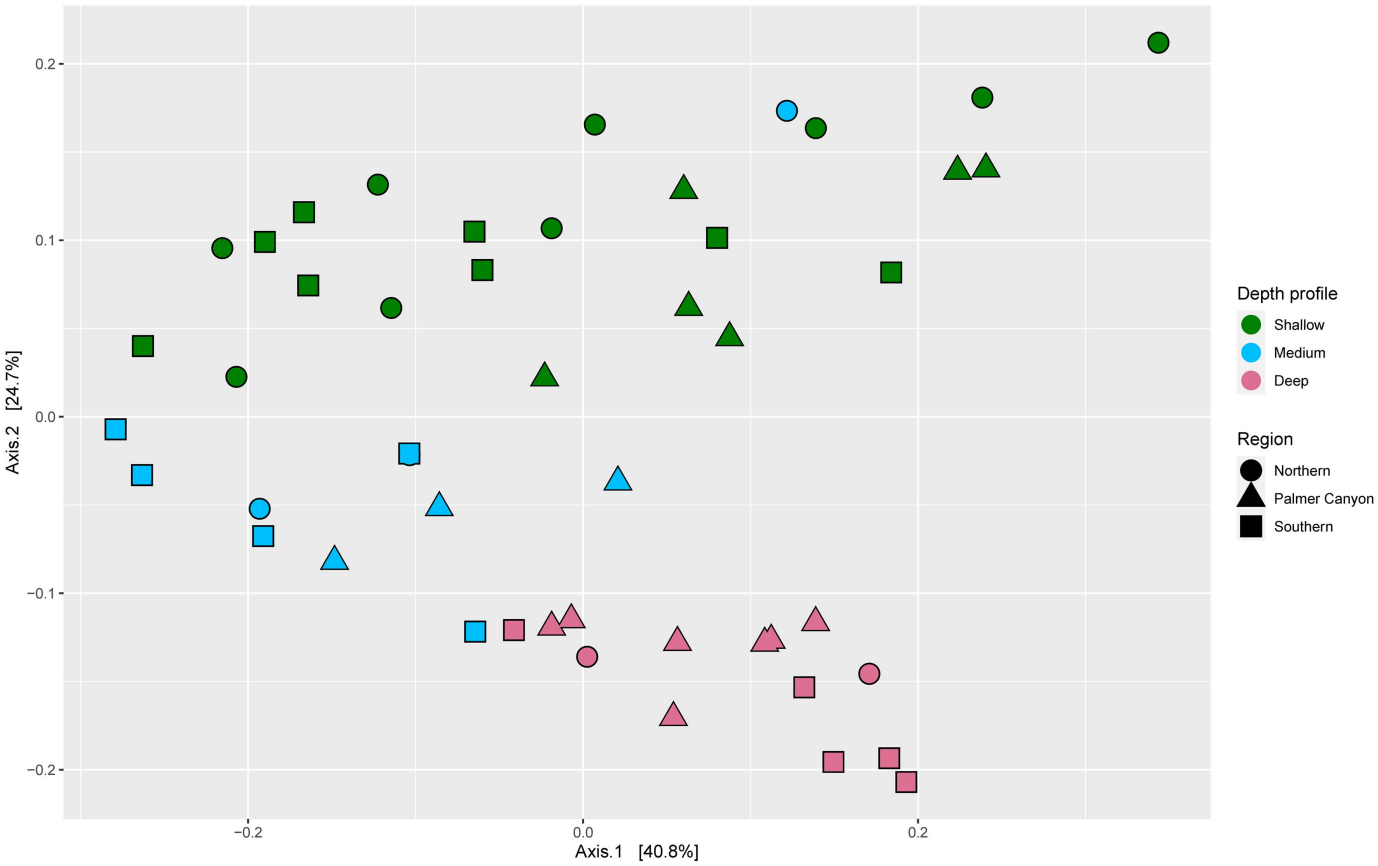
Principal coordinates analysis (PCoA) of Bray-Curtis dissimilatory based on normalized gene coverage across 48 samples. Symbol shape and color indicates sample region and depth, respectively.

Microbial community functions varied across depths (Figure 2). Three distinct clusters were observed for deep, medium-deep, and shallow samples with minor overlaps based on normalized gene coverage (PERMANOVA, *p* = 0.001). One of the medium-deep samples (Armstrong Reef) clustered with the shallower-depth samples. Dispersion among sample groups differed according to depth (*p* = 0.001). The average distance from the median was highest for shallower depth samples, followed by medium-deep and deeper samples. The samples were also significantly different (PERMANOVA, *p* = 0.011) based on region (Southern, Northern, and Palmer Canyon). However, the separation of samples was more pronounced for depth than region according to the PERMANOVA F-ratio (F_depth_ = 10.224; *p* = 0.001 and F_region_ = 2.6985; *p* = 0.011). We conducted a detailed study of carbon, nitrogen, sulfur, and carbohydrate metabolism-specific genes based on normalized gene coverage to understand the differential abundances of functional genes across different depth horizons and regions.

#### 3.1.1. Carbon cycle

**Figure 3:**
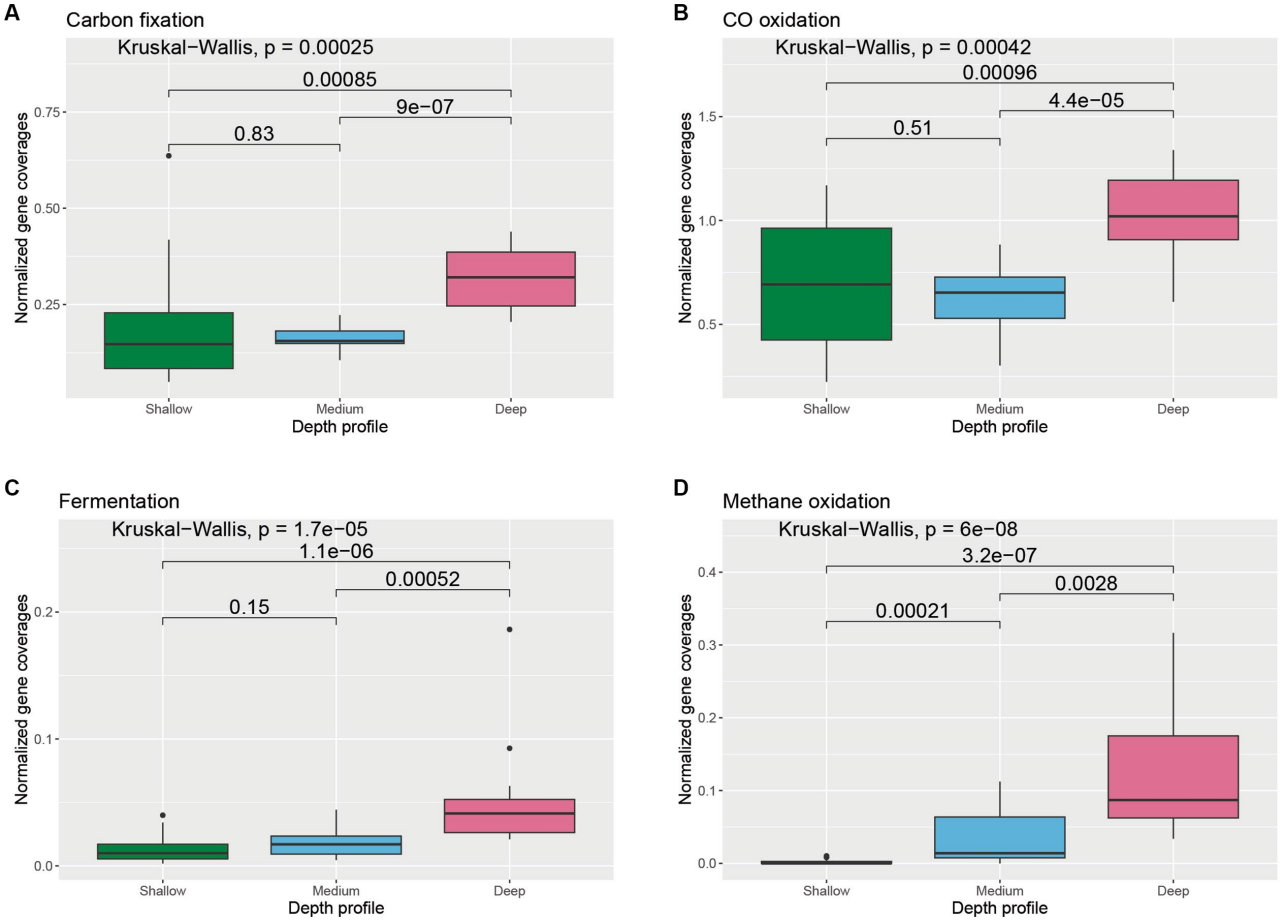
Boxplot showing the distribution of different carbon cycle genes related to **(A)** carbon fixation, **(B)** CO oxidation, **(C)** fermentation, and **(D)** methane oxidation across different depth horizons of the wAP. Normalized coverages of genes grouped in each of these categories are listed in Supplementary Figure S1. A pairwise comparison for significance was conducted using the Wilcoxon test.

Key genes involved in prokaryotic dark carbon fixation, carbon monoxide oxidation, fermentation, methane oxidation, and photoheterotrophy were explored to understand carbon transformation and their distribution in the wAP. Varied abundances of groups of genes involved in different carbon transformation processes were observed across different depths (Supplementary Figure S1). Genes associated with dark carbon fixation were found to be significantly higher in the deeper samples compared to medium-depth (*p* = 9 × 10^−7^) and shallower samples (*p* = 0.00085) (Figure 3A). The abundance of the genes involved in CO oxidation varied across depths. The deeper samples harbored significantly greater CO oxidation gene coverages compared to shallower (*p* = 0.00096) and medium-deep samples (*p* = 4.4 × 10^−5^) (Figure 3B). A similar trend was observed for genes involved in fermentation and methane metabolism (Figures 3C,D).

#### 3.1.2. Sulfur cycle

**Figure 4:**
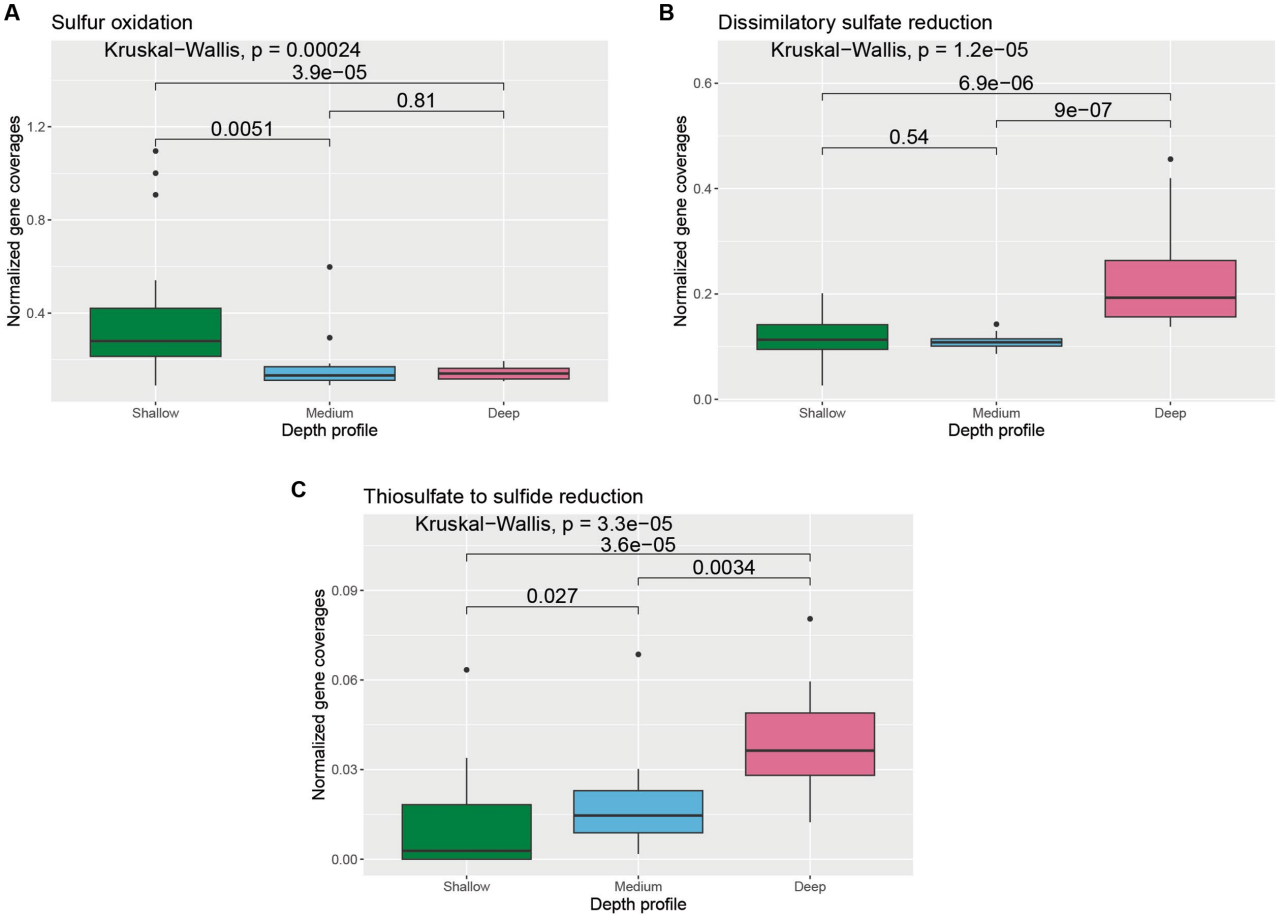
Boxplot showing the distribution of different sulfur cycle genes related to **(A)** sulfur oxidation, **(B)** dissimilatory sulfate reduction, and **(C)** thiosulfate to sulfide reduction across different depth horizons of the wAP. Normalized coverages of genes grouped in each of these categories are listed in Supplementary Figure S2. A pairwise comparison for significance was conducted using the Wilcoxon test.

The abundance of genes involved in the oxidation and reduction of sulfur species varied across depths (Supplementary Figure S2). Normalized coverages of genes involved in sulfur oxidation were found to be significantly higher in the shallower samples compared to medium-depth (*p* = 0.0051) and deeper samples (*p* = 3.9 × 10^−5^) (Figure 4A). On the contrary, normalized coverage of genes involved in dissimilatory sulfate reduction was found to be significantly higher in the deeper samples compared to medium-depth (*p* = 9 × 10^−7^) and shallower samples (*p* = 6.9 × 10^−6^) (Figure 4B). Normalized coverage of genes involved in thiosulfate to sulfide reduction was found to be the highest in the deeper samples, followed by medium-depth and shallower samples (Figure 4C).

#### 3.1.3. Nitrogen cycle

**Figure 5:**
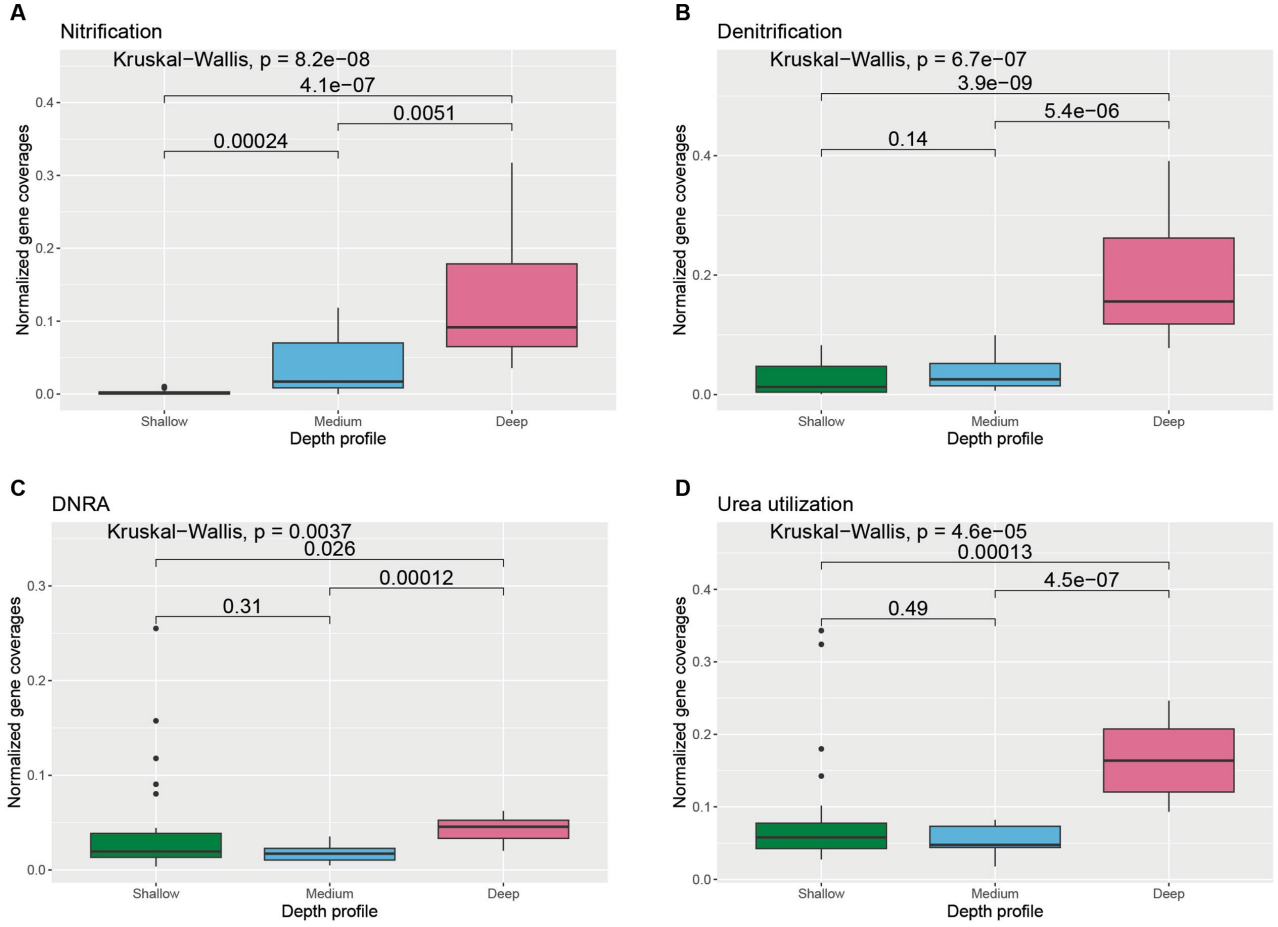
Boxplot showing the distribution of different nitrogen cycle genes related to **(A)** nitrification, **(B)** denitrification, **(C)** dissimilatory nitrate reduction to ammonia (DNRA), and **(D)** urea utilization across different depth horizons of the wAP. Normalized coverages of genes grouped in each of these categories are listed in Supplementary Figure S3. A pairwise comparison for significance was conducted using the Wilcoxon test.

The abundances of the genes involved in oxidation and reduction of nitrogen species also varied with depth (Supplementary Figure S3). The average coverages of the genes involved in nitrification were significantly higher in the deeper horizons of the water column compared to medium-depth (*p* = 0.0051) and shallower horizons (*p* = 4.1 × 10^−7^) (Figure 5A). Similarly, the abundance of genes involved in denitrification and dissimilatory nitrate reduction to ammonia (DNRA) was found to be significantly higher in deeper samples compared to medium-deep (Denitrification: *p* = 5.4 × 10^−6^; DNRA: *p* = 0.00012) and shallower depth (Denitrification: *p* = 3.9 × 10^−9^; DNRA: *p* = 0.026) samples (Figures 5B,C). Genes involved in urea utilization were also observed in all the wAP samples. Urea-utilizing gene coverages were found to be significantly higher in the deeper horizon samples compared to samples from the medium depth (*p* = 4.5 × 10^−7^) and shallower depth (*p* = 0.00013) horizons (Figure 5D).

#### 3.1.4. Carbohydrate transformations

**Figure 6:**
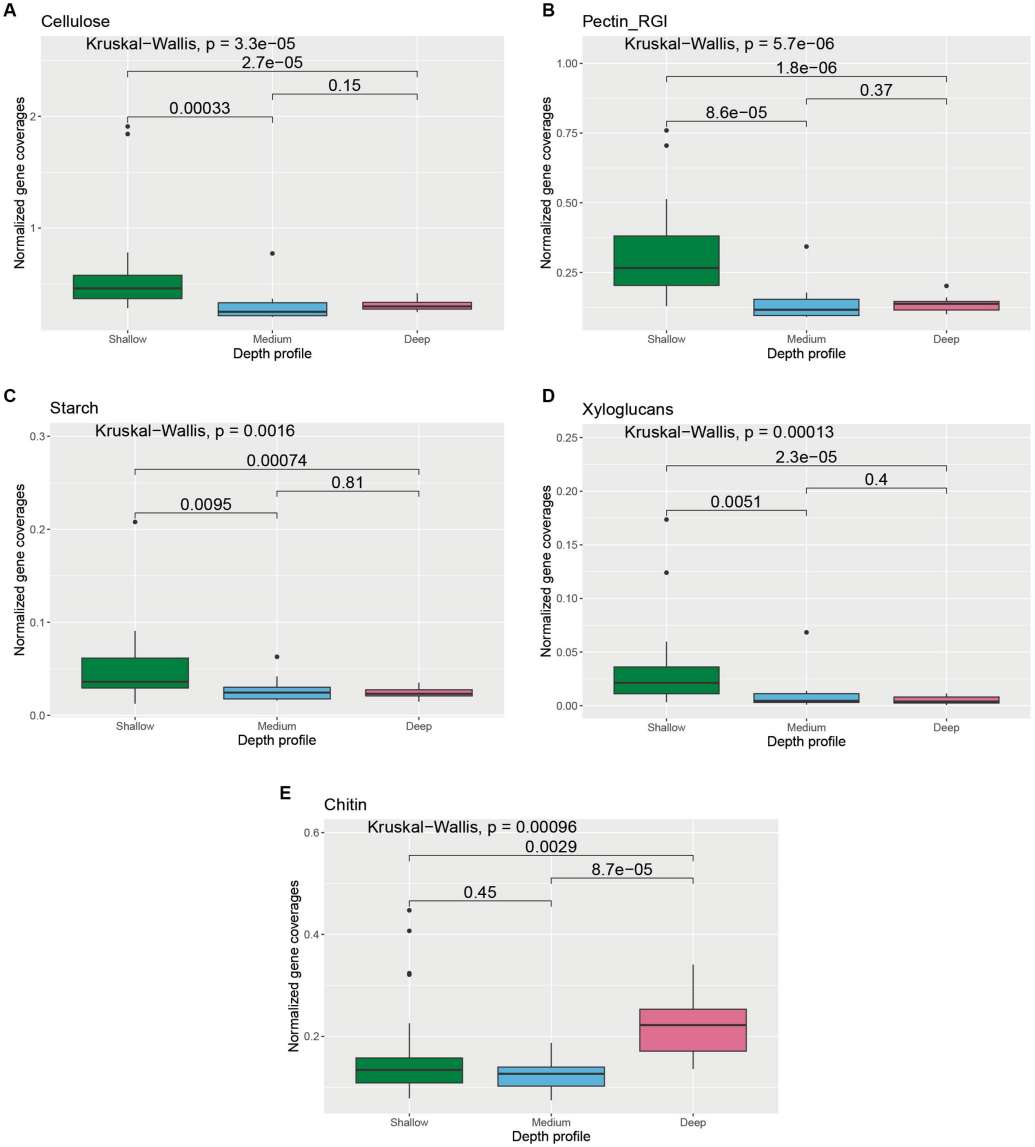
Boxplot showing the distribution of different carbohydrate transformation genes related to **(A)** cellulose, **(B)** pectin RGI, **(C)** starch, **(D)** xyloglucan, and **(E)** chitin degradation across different depth horizons of the wAP. Normalized coverages of genes grouped in each of these categories are listed in Supplementary Figure S4. A pairwise comparison for significance was conducted using the Wilcoxon test.

We further analyzed genes involved in the transformation of glycoprotein, cellulose, chitin, pectin, starch, xylans, and xyloglucans to understand the carbohydrate pool and transformation capabilities of microorganisms across different depth horizons of the wAP (Supplementary Figure S4). Cellulose, pectin (RGI), starch, and xyloglucans metabolizing gene coverages were found to be significantly higher in the shallower depth samples compared to medium-deep [Cellulose: *p* = 0.00033; pectin (RGI): *p* = 8.6 × 10^−5^; starch: *p* = 0.0095; and xyloglucans: *p* = 0.0051] and deeper samples [cellulose: *p* = 2.7 × 10^−5^; pectin (RGI): *p* = 1.8 × 10^−6^; starch: *p* = 0.00074; and xyloglucans: *p* = 2.3 × 10^−5^] (Figures 6A–D). Abundances of chitin metabolizing genes were significantly higher in deeper samples compared to the samples from medium depth (*p* = 8.7 × 10^−5^) and shallow samples (*p* = 0.0029) (Figure 6E).

### 3.2. Distribution, taxonomy, and metabolic profiles of MAGs

**Figure 7:**
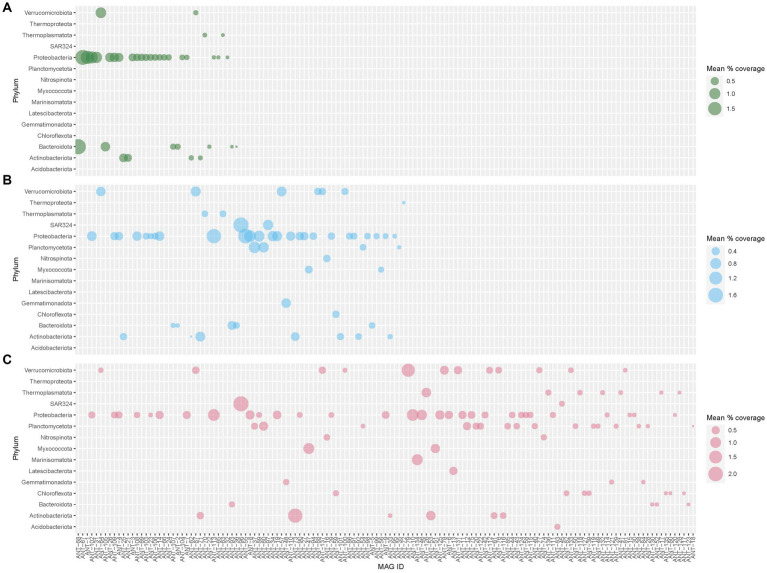
Average abundance of 137 dereplicated MAGs across **(A)** shallow, **(B)** medium, and **(C)** deep waters of the coastal wAP. Each dereplicated MAG represents multiple MAGs from different samples. The average abundance of each dereplicated MAG represents the average abundance of all the MAGs it represents from a particular depth horizon.

A total of 2,940 bins were obtained from 48 samples. Of these, 612 bins with genome completeness of more than 70% and contamination of less than 5% were considered MAGs and used for further analysis. These 612 MAGs were filtered down to 609 MAGs by dRep based on genome quality and were dereplicated to a final set of 137 MAGs. 137 MAGs (representing 609 MAGs) covered 13.11, 5.96, and 2.12% of the total filtered reads from deep, medium-deep, and shallow samples, respectively (Figure 7). Among these 137 MAGs, 64 MAGs were unique to deep samples, whereas 19 and 15 MAGs were unique to the medium and shallow horizons, respectively. 11 MAGs were found in all three depth horizons, and the remaining 28 MAGs were found in two of the three horizons (medium and deep: 18, shallow and medium: 9, and shallow and deep: 1). When sorted by region, 33 MAGs represented MAGs unique to the Southern region, whereas 32 and 4 MAGs represented MAGs unique to the Palmer Canyon and Northern region, respectively. 21 MAGs were found in all three regions, and the remaining 47 MAGs were found in two of the three regions (Southern—Palmer Canyon: 30, Southern—Northern: 14, and Northern—Palmer Canyon: 3). The most abundant MAG found in the shallower waters was affiliated with Bacteroidota (represented by ANT-68) (Figure 7A). This MAG was exclusively observed in the shallow environments of the coastal wAP. The most abundant MAG found in the medium-depth waters was affiliated with family SAR324 (represented by ANT-96) (Figure 7B). This MAG was observed in the medium-depth and deeper samples. ANT-96 was also found to be the most abundant MAG in deeper waters (Figure 7C). Considering the proportion of reads that mapped to each MAG, the overall highest average mapped read percentage was also found in ANT-96 (2.11% ± 1.22%; representing 7 MAGs from different samples). Detailed genome statistics of the 137 MAGs are present in Supplementary Table S3.

**Figure 8:**
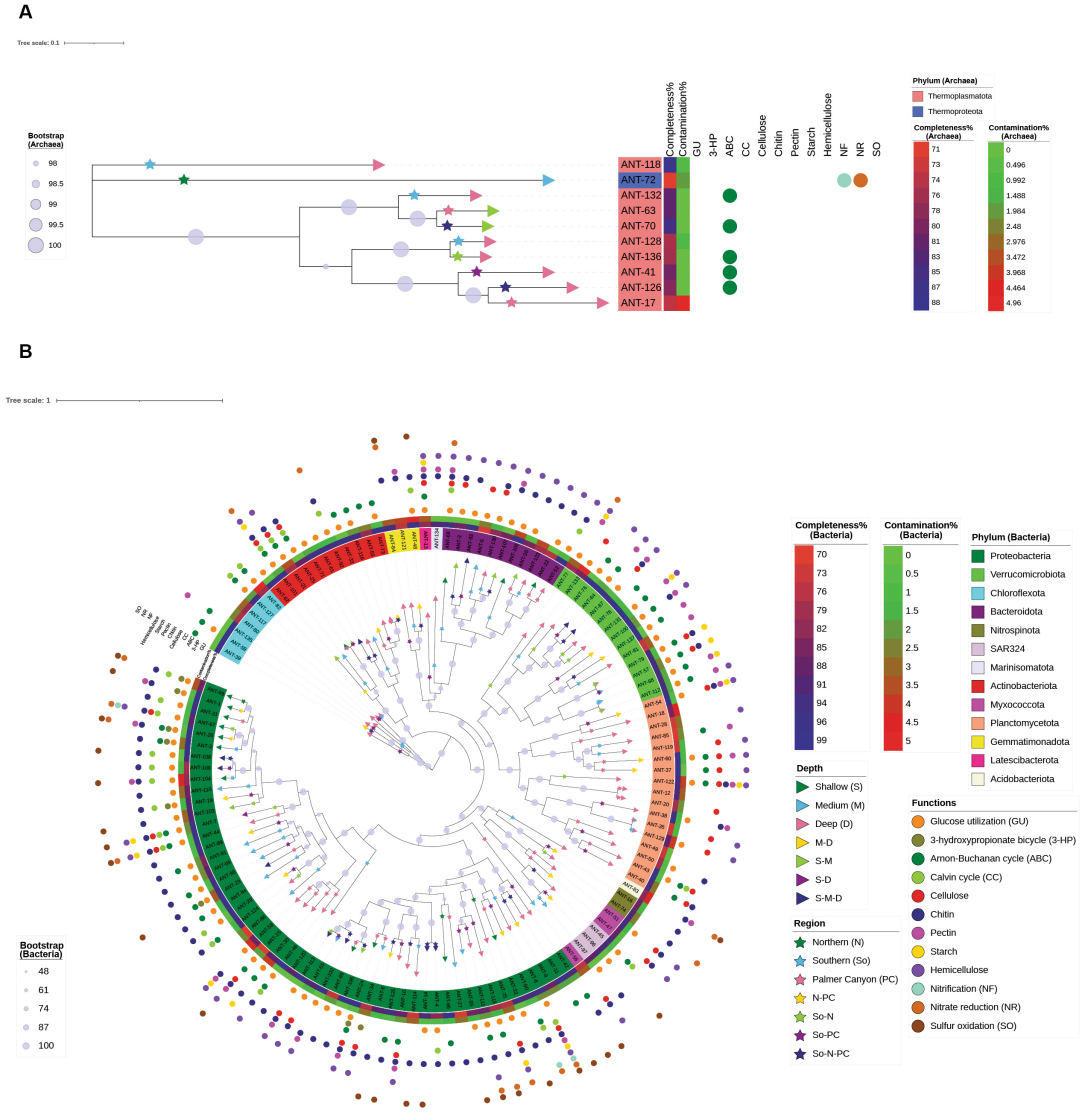
Maximum-likelihood phylogenomic analysis of 137 dereplicated MAGs based on **(A)** archaeal and **(B)** bacterial marker genes from GTDB-Tk analysis. Detailed genome statistics and taxonomy are provided in Supplementary Tables S3, S4, and the detailed pathways and functions are provided in Supplementary Table S5. Phylum, completeness %, contamination %, and bootstrap legends are domain-specific, whereas depth, region, and function are common for bacteria and archaea. Depth (represented by a right triangle) and region (represented by a star) symbols are present on the terminal node and terminal branch, respectively. The circle symbols in the outermost layer depict the functional capabilities of each MAG. All the carbohydrates mentioned represent the presence of enzymes capable of degrading them. Xylan and/or xyloglucan degrading enzymes are represented by hemicellulose in this figure. The hyphenated legends for depth represent MAGs found in multiple depth horizons, whereas hyphenated legends for regions represent MAGs found in multiple regions.

The MAGs were taxonomically diverse. Of 137 MAGs, 10 MAGs were affiliated with the domain Archaea, and 127 MAGs were affiliated with Bacteria. Average nucleotide identity (ANI) or relative evolutionary divergence (RED) values were analyzed for each MAG based on reference genomes from GTDB using GTDB-Tk. RED values were calculated when the MAGs were unable to be classified based on ANI. ANI values greater than 0.95 were obtained for 40 MAGs (2 archaeal MAGs and 38 bacterial MAGs), whereas RED values (ranging between 0.660 to 0.998) were obtained for the rest of the MAGs (Supplementary Table S4). Nine of the archaeal MAGs were affiliated with the phylum Thermoplasmatota whereas one was affiliated with Thermoproteota (Figure 8A). Among the ten archaeal MAGs, two MAGs had formal taxonomic nomenclature (as indicated in the International Code of Nomenclature of Prokaryotes) at the genus level (*Nitrosopumilus* and *Thalassarchaeum*), and one of the Thermoplasmatota MAGs had a separate branch from the root of the phylogenomic tree. Bacterial MAGs were assigned to 13 different phyla (Figure 8B). The highest number of MAGs were affiliated with Proteobacteria (53 MAGs), followed by 17 and 14 MAGs affiliated with Planctomycetota and Verrucomicrobiota, respectively. Out of 127 bacterial MAGs, 61, 28, and 5 MAGs had a formal taxonomic nomenclature at the family, genus, and species levels, respectively. Two bacterial MAGs affiliated with class Alphaproteobacteria (ANT-120) and phylum Planctomycetota (ANT-49) had RED values lower than 0.70. Detailed taxonomy classifications of all 137 MAGs are present in Supplementary Table S4. It was interesting to note that one of the MAGs classified as Myxococcota by GTDB-Tk clustered with Proteobacterial MAGs on the phylogenomic tree (Figure 8B).

**Figure 9:**
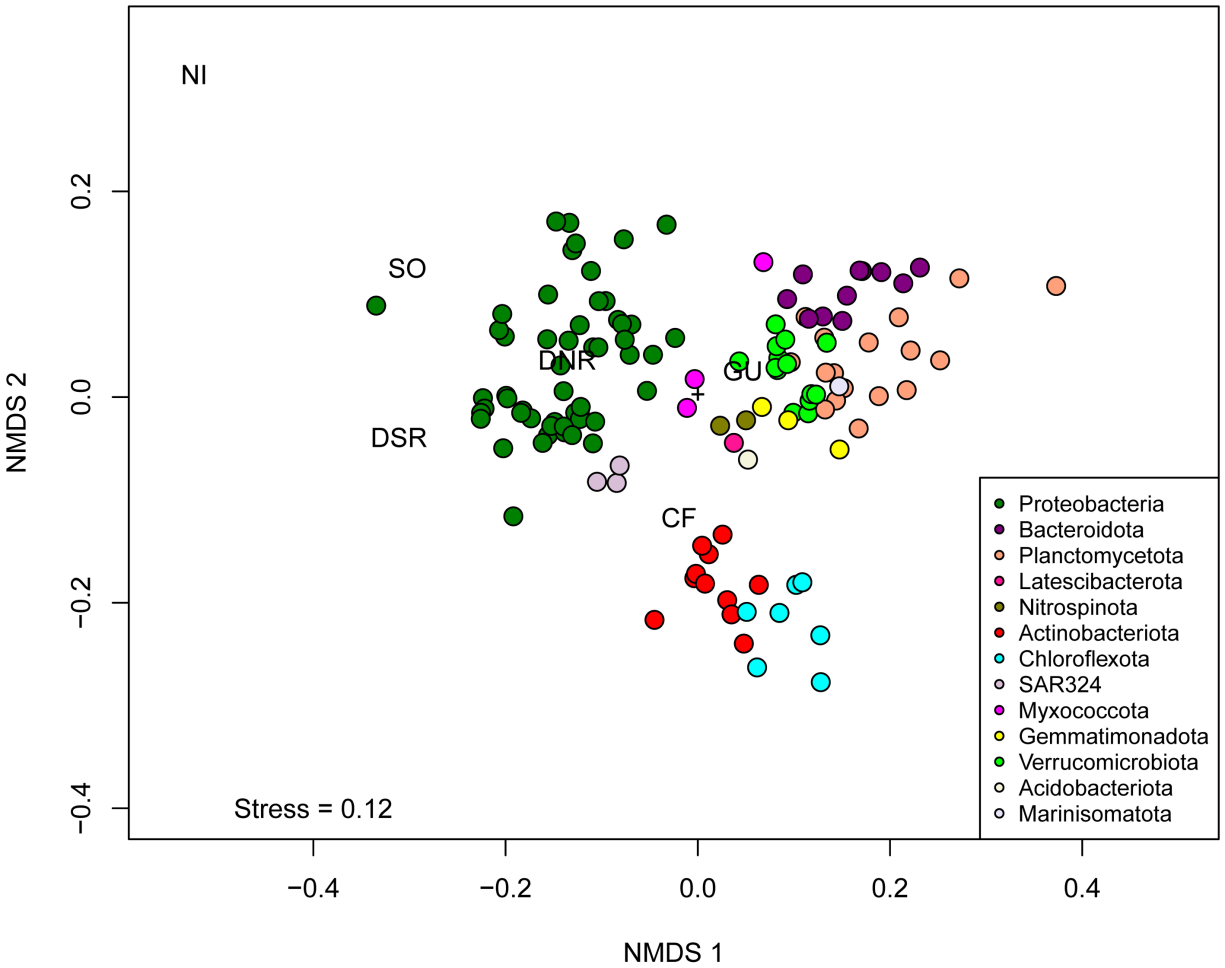
Non-Metric Multidimensional Scaling (NMDS) of binary matrix based on the presence or absence of genes predicted by Ghost Koala across 127 dereplicated bacterial MAGs. Abbreviations of metabolic categories as used in the NMDS analysis are as follows: GU, Glucose utilization; CF, carbon fixing pathways; NI, nitrification; SO, sulfur oxidation; DNR, Denitrification; DSR, Dissimilatory sulfate reduction.

Distinct groups of MAGs were observed when clustered according to their metabolic profiles. The NMDS plot indicated that the metabolic profiles of bacterial populations were phylum-specific, with minor overlaps among phyla (Figure 9). Distinct clusters for archaeal and bacterial MAGs were observed on the PCoA plot based on genomic repertoire (PERMANOVA, *p* = 0.001) (Supplementary Figure S5). Specific gene sets, and pathways were studied to determine the metabolic capabilities of the MAGs (Supplementary Table S5 and Supplementary Figure S6).

#### 3.2.1. Role of MAGs in carbon transformation

Capabilities of glucose utilization and carbon fixation were analyzed based on different pathways. Since these pathways have multiple enzymes involved in them, pathways having ≥70% completeness in a MAG were considered as the presence of the pathway in the MAG. The details of the pathways and the completeness profiles are described in Supplementary Table S5. The capability to use externally fixed carbon was studied based on the presence of the Embden-Meyerhof and Entner–Doudoroff pathways. These two pathways help in the conversion of glucose into pyruvate. 75 MAGs covering 10 phyla had the capability of performing the Embden Meyerhof pathway, whereas 42 MAGs covering seven phyla had genomic repertoires for the Entner–Doudoroff pathway. The pentose phosphate pathway was observed in 88 MAGs covering 11 phyla. The citric acid cycle (Krebs cycle) was observed in 112 MAGs, whereas the glyoxylate cycle was observed in 40 MAGs. Three carbon fixation pathways *viz.* 3-Hydroxypropionate bicycle, Arnon–Buchanan cycle (reductive citrate cycle), and Calvin cycle (reductive pentose phosphate cycle) were observed among the MAGs. Capabilities of the Arnon–Buchanan cycle were present in a higher number of MAGs (56 MAGs) compared to the Calvin cycle (present in 26 MAGs) and 3-Hydroxypropionate bicycle (present in 8 MAGs). 3-Hydroxypropionate bicycle was only present in Proteobacterial MAGs, whereas Arnon–Buchanan cycle was distributed over MAGs affiliated to Proteobacteria, Verrucomicrobiota, Actinobacteriota, Planctomycetota, Chloroflexota, Thermoplasmatota, SAR324, and Latescibacterota. Calvin cycle was present in MAGs affiliated to Proteobacteria, Actinobacteriota, Chloroflexota, Bacteroidota, Planctomycetota, Gemmatimonadota, and SAR324.

The ability to produce or catabolize small-chain fatty acids and alcohols was studied based on the presence of certain genes in the genomic inventories of the MAGs. Capabilities of alcohol production (EC 1.1.1.1) were found in 53 MAGs, with the majority of the MAGs affiliated with Proteobacteria. The presence of genes encoding for phosphate acetyltransferase (EC 2.3.1.8) and/or acetate kinase (EC 2.7.2.1) was studied to understand acetate metabolism in the MAGs. Genes encoding for acetate metabolizing enzymes were found in 21 MAGs which were affiliated with six different phyla (Proteobacteria, Verrucomicrobiota, Planctomycetota, Actinobacteriota, Latescibacterota, and Bacteroidota). Genes encoding for L-lactate metabolizing enzyme (L-lactate dehydrogenase) were found in 19 MAGs, which were affiliated with the Proteobacteria, Actinobacteriota, Verrucomicrobiota, and Planctomycetota, whereas genes encoding for D-lactate metabolizing enzyme (D-lactate dehydrogenase) were found in five MAGs affiliated with Proteobacteria, Verrucomicrobiota, and Bacteroidota. The gene encoding for propionate metabolism (propionate CoA transferase) was found in three MAGs affiliated with two different phyla (Proteobacteria and Verrucomicrobiota).

Genes encoding for carbohydrate-active enzymes were studied to understand the polysaccharide-degrading capabilities of the MAGs. Genes encoding for enzymes involved in breaking down amorphous cellulose were found in 30 MAGs affiliated with seven different phyla (Proteobacteria, Planctomycetota, Bacteroidota, Myxococcota, Verrucomicrobiota, Actinobacteriota, and Latescibacterota), whereas genes encoding for the enzyme involved in breaking down crystalline cellulose was found in 11 MAGs covering five phyla (Planctomycetota, Proteobacteria, Verrucomicrobiota, Actinobacteriota, and Myxococcota). Genes coding for chitin-degrading enzymes were found in 85 MAGs (found in all phyla detected in this study except for MAGs affiliated with Thermoproteota, Chloroflexota, and Thermoplasmatota), whereas genes for starch-degrading enzymes were found in 15 MAGs having affiliation with five phyla (Proteobacteria, Verrucomicrobiota, Planctomycetota, Actinobacteriota, and Latescibacterota). Genes encoding enzymes that can perform xylan and xyloglucan (major components of hemicellulose) degradation were found in 23 and 40 MAGs, respectively. MAGs with the ability to degrade xylan were affiliated with Proteobacteria, Bacteroidota, Myxococcota, Planctomycetota, Actinobacteriota, and Latescibacterota, whereas MAGs with the ability to degrade xyloglucan were affiliated to Proteobacteria, Bacteroidota, Verrucomicrobiota, Planctomycetota, Myxococcota, Latescibacterota, and Marinisomatota. Genes encoding pectin degradation enzymes were found in 31 MAGs (having affiliation with Proteobacteria, Verrucomicrobiota, Planctomycetota, Bacteroidota, Latescibacterota, Marinisomatota, Gemmatimonadota, Acidobacteriota, and Chloroflexota).

#### 3.2.2. Role of MAGs in sulfur and nitrogen transformation

Genes involved in thiosulfate oxidation were found in 26 MAGs having affiliations with four phyla (Proteobacteria, Gemmatimonadota, Acidobacteriota, and SAR324) (Supplementary Figure S6). Three proteobacterial MAGs were found to harbor genes for dissimilatory sulfate reduction.

Genes involved in ammonia oxidation were found in two proteobacterial MAGs and in one archaeal MAG (affiliated to Thermoproteota). Genes involved in the conversion of nitrite to nitrate (one of the steps in nitrification) were found in two proteobacterial MAGs. Genes involved in the conversion of nitrate to nitrite (one of the steps in denitrification or dissimilatory nitrate reduction to ammonia) were found in two proteobacterial MAGs. Enzymes involved in the conversion of nitrite to nitric oxide (a key step in denitrification) were found in 14 different MAGs covering eight phyla (Proteobacteria, Actinobacteriota, Thermoproteota, Gemmatimonadota, Verrucomicrobiota, Acidobacteriota, Bacteroidota, and Nitrospinota), whereas enzymes involved in the conversion of nitric oxide to nitrous oxide (an additional step in denitrification) were found in two proteobacterial MAGs.

### 3.3. Putative metabolic lifestyles of MAGs

**Figure 10:**
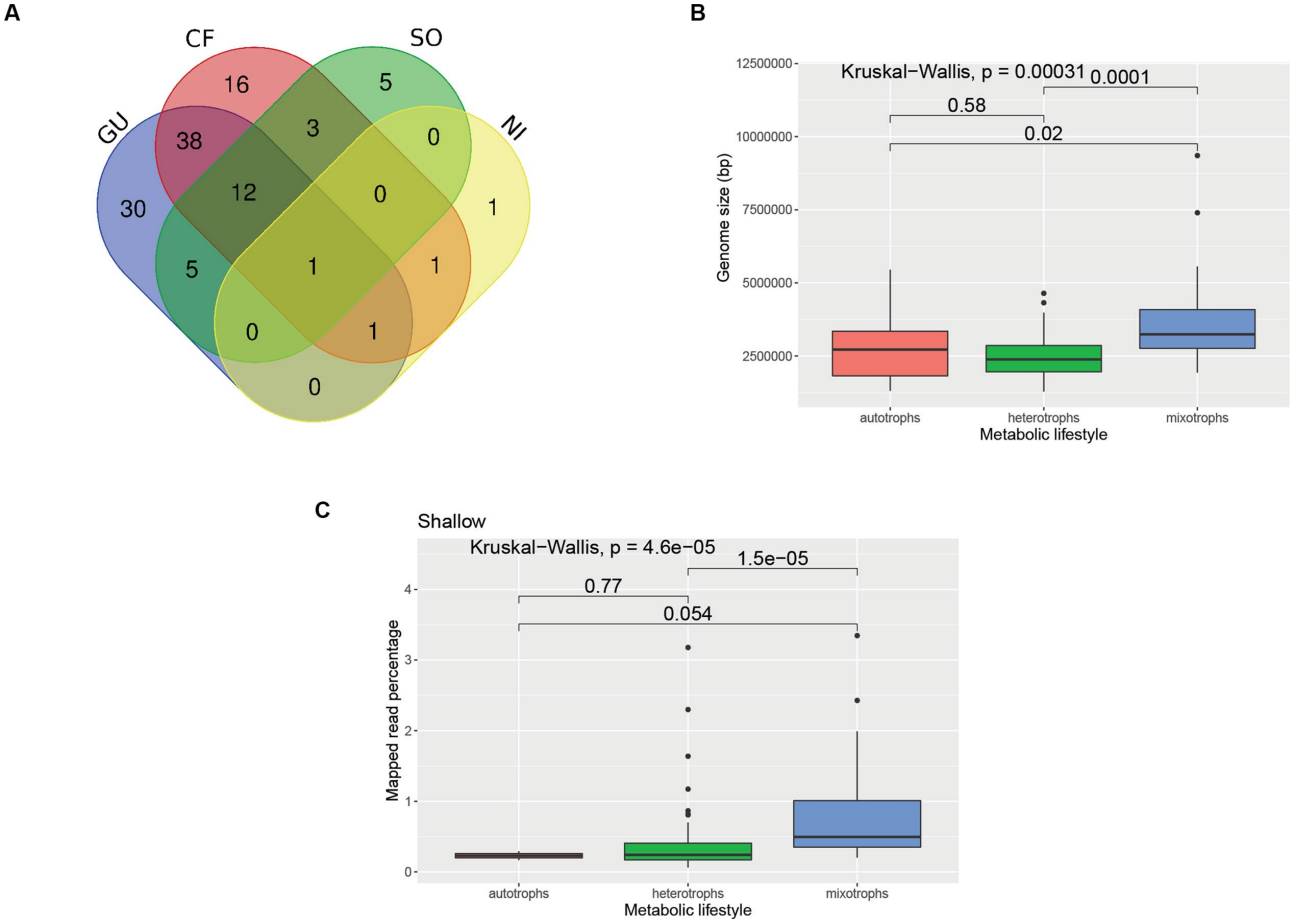
Comparison of MAGs having different metabolic lifestyles. **(A)** Venn Diagram showing overlaps among different metabolic categories of the MAGs. The numbers depicted in the Venn diagram represent the number of MAGs affiliated to each category. GU, Glucose utilization; CF, carbon fixing pathways; NI, nitrification; SO, sulfur oxidation. **(B)** Boxplot showing genome sizes of MAGs related to different metabolic lifestyles. The details of the genome sizes and metabolic lifestyle of each MAG are reported in Supplementary Table S3. **(C)** Boxplot showing mapped read percentage of MAGs having different metabolic lifestyles in shallower depth samples. For the boxplots, a pairwise comparison for significance was conducted using the Wilcoxon test.

Overlaps across different metabolic categories are reported in Figures 8, 10A. It was found that the average genome sizes of the mixotrophs were significantly higher compared to heterotrophs (*p* = 0.0001) and autotrophs (*p* = 0.02) (Figure 10B). In the shallower depth samples, a significantly high mapped read percentage was observed in the mixotrophic MAGs (*n* = 37 MAGs), while compared to the autotrophic (*n* = 2 MAGs) and heterotrophic MAGs (*n* = 52 MAGs) (mixotroph vs. autotrophs, *p* = 0.054; mixotroph vs. heterotroph, *p* = 1.5 × 10^−5^) (Figure 10C). In the medium and deeper samples, the mapped read percentages for mixotrophic, autotrophic, and heterotrophic MAGs were not significantly different.

#### 3.3.1. Putative mixotrophs

Four categories of mixotrophs covering 52 dereplicated MAGs were obtained (Supplementary Figure S6 and Supplementary Table S5). Only one MAG (ANT-6) recovered from a deeper horizon sample and classified as *Paraburkholderia* was found to harbor genes from all four categories of metabolism (GU, CF, SO, and NI). There were 12 MAGs that harbored genes from GU, CF, and SO (11 affiliated to Proteobacteria and one affiliated to Gemmatimonadota), whereas there was only one MAG that harbored genes from GU, CF, and NI (affiliated to Proteobacteria). There were 38 MAGs that harbored the genes from GU and CF metabolism categories only (affiliated to Proteobacteria, Actinobacteriota, Verrucomicrobiota, Planctomycetota, Chloroflexota, Bacteroidota, Latescibacterota, and SAR324).

#### 3.3.2. Putative autotrophs

Three categories of autotrophs covering 20 dereplicated MAGs were obtained (Supplementary Table S5). There were three MAGs harboring genes for CF and SO (affiliated with Gammaproteobacteria), whereas there was only one MAG harboring gene for CF and NI (affiliated with Gammaproteobacteria). There were 16 MAGs that had genomic repertoire for CF but lacked genes for SO and NI (affiliated with Proteobacteria, Thermoplasmatota, Planctomycetota, Chloroflexota, Actinobacteriota, and Verrucomicrobiota).

#### 3.3.3. Putative heterotrophs

Two categories of heterotrophs covering 35 dereplicated MAGs were observed in this study (Supplementary Figure S6 and Supplementary Table S5). One of the categories harbored genes for GU and SO, which was found in five different MAGs (affiliated to Proteobacteria and SAR324). The other category solely harbored genes for GU and was found in 30 MAGs (affiliated to Proteobacteria, Bacteroidota, Verrucomicrobiota, Planctomycetota, Actinobacteriota, SAR324, and Myxococcota).

## 4. Discussion

Our analysis reveals the diverse genomic repertoire contained among marine bacteria and archaea along the coastal wAP. PCoA based on normalized gene coverage across 48 samples suggested that microbial community functions were strongly partitioned by depth, with the highest variation in microbial community function observed in the shallower samples (0–40 m). A possible explanation is that this higher variance results from a more dynamic environment in the shallower waters than in the deeper environments (Carranza et al., 2018).

Primary production in the coastal Antarctic is attributed primarily to phytoplankton (Arrigo et al., 2008). However, prokaryotic dark carbon fixation can be significant in deep and polar oceans (Alonso-Sáez et al., 2010; Williams et al., 2012; Connelly et al., 2014), and previous work has identified the genomic signatures of dark carbon fixation along the wAP (Grzymski et al., 2012). We observed genes indicative of dark carbon fixation in numerous MAGs representing multiple phyla. This suggests that in addition to eukaryotic carbon fixation, prokaryotic dark carbon fixation can also be a source of fixed carbon in the Antarctic marine ecosystem, expanding the conventional viewpoint of marine primary production (Buchan et al., 2014). We are not aware of any studies that attempt to quantify dark carbon fixation inputs to coastal Antarctic ecosystems. However, previous work suggests oceanic primary production estimates would increase by 5 –22% when total dark DOC fixation is included (Baltar and Herndl, 2019). Moreover, the normalized coverage of genes related to dark carbon fixation was found to be higher in deeper waters compared to the waters from medium-deep and shallower horizons in the coastal wAP suggesting that the dearth of phytoplankton-fixed carbon in the deeper waters selects for microorganisms capable of fixing carbon in the dark. This lack of photosynthate as an electron donor in deeper water is further supported by the presence of higher normalized gene coverage of CO oxidation genes. Lappan et al. (2023) previously reported an enrichment of CO hydrogenase (an enzyme involved in CO oxidation) in temperate mesopelagic waters and suggested that CO oxidation is favored in energy-limited waters at depths where primary production is low.

The normalized gene coverages for processes preferred in hypoxic/anoxic environments, such as fermentation, dissimilatory sulfate reduction, thiosulfate to sulfide reduction, denitrification, and DNRA, were found to be higher in the water samples from the deeper horizons (>100 m). Although oxygen drawdown is observed below the photic zone in the wAP (Cape et al., 2019), the water column typically remains sufficiently oxic to support aerobic processes, including nitrification and methane oxidation in deeper waters. We suggest two explanations for the presence of anaerobic pathways here. First, the organisms harboring these pathways may be facultative anaerobes that can dwell in multiple environments of the wAP. This is supported by the presence of certain MAGs capable of using oxygen (presence of cytochrome c oxidase and F-Type ATPases genes) and nitrate (presence of genes involved in denitrification) as terminal electron acceptors. Second, these microbial populations may come from fecal pellets and sinking detritus that harbor microenvironments that support the growth of anaerobic microbial populations.

Due to high levels of primary production, the coastal wAP harbors a diverse and abundant carbohydrate pool. The source of these carbohydrates includes phytoplankton or zooplankton such as krill (van Oijen et al., 2003; Yu et al., 2020). We observed a diverse genomic repertoire for degrading carbohydrates derived from phytoplankton and zooplankton. This suggests a link between the microeukaryotic and prokaryotic populations in the Antarctic. Moreover, higher abundances of microorganisms capable of degrading cellulose, xyloglucans, pectin, and starch were observed in the upper water column, consistent with the greater phytoplankton biomass expected there. This distribution suggests that carbohydrates are readily turned over in the upper water column, possibly reducing vertical export. This is further supported by the presence of prokaryotic dark carbon fixing pathways in the deeper environment of coastal wAP. Several MAGs having the capability to degrade polysaccharide was also observed in the medium-deep and deeper horizon of the wAP. This suggests that these microbial populations may be associated with the sinking detritus material, which helps in the conversion of complex carbohydrates to simpler carbon compounds that can be used by other heterotrophs.

Varied metabolic flexibility was observed in several MAGs. ANT-6, affiliated to *Paraburkholderia fungorum* was found to have the most diverse genomic repertoire based on the presence of heterotrophic and autotrophic pathways. The genomic repertoire suggests that they can perform carbon fixation and also utilize glucose. The mixotrophic behavior of *Paraburkholderia* has been reported previously (Herpell et al., 2020). Interestingly, ANT-6 also harbored genes involved in thiosulfate oxidation, nitrification, and denitrification. Sulfite oxidation was previously observed in *Paraburkholderia caledonica* PHRS4 (BioCyc ID: PWY-5276). Even though varied metabolic traits of the genus *Paraburkholderia* are well known, the presence of genomic machinery related to autotrophic and heterotrophic lifestyle, along with the presence of thiosulfate oxidation, nitrification, and denitrification capability in a single strain, is not reported elsewhere to the best of our knowledge. The genome size of ANT-6 was found to be the highest (9.3 Mbp) among all the other MAGs constructed. The genome size of ANT-6 is in the range of genome sizes of other *Paraburkholderia* strains (Wang et al., 2021), which is bigger than “typical” bacterial genomes (Land et al., 2015). The metabolic flexibility of *Paraburkholderia* is consistent with its bigger genomes size. Previous reports of the presence of *Paraburkholderia* in the Antarctic environment (Malard et al., 2022) also support the presence of ANT-6 in the Antarctic.

A high number of mixotrophic MAGs (52 MAGs) were observed compared to exclusively heterotrophic (35) and autotrophic (20) MAGs. The prevalence of mixotrophy was also previously reported in Arctic prokaryotic genomes (Royo-Llonch et al., 2021). In the shallow samples of the coastal wAP, the abundances of mixotrophic MAGs were found to be significantly higher compared to heterotrophic and autotrophic MAGs, whereas, in the deeper samples, the abundances of the mixotrophic MAGs were not significantly different compared to autotrophic and heterotrophic MAGs. This suggests a strong pressure for metabolic flexibility, potentially a response to the seasonal boom-bust cycle of photosynthetic primary production. This is in line with the higher variations of genomic repertoire in the shallower samples compared to medium-depth and deeper samples. We hypothesize that dynamic environments select microbial populations with a diverse genomic repertoire, including the capacity to switch between heterotrophic and autotrophic lifestyles. Moreover, the genome size of the mixotrophs was found to be significantly larger compared to the autotrophs and heterotrophs. This is in accordance with a previous study where they reported larger genome sizes of the generalist compared to the specialist (Sriswasdi et al., 2017). However, it should be taken into account that higher completeness of MAGs is required to ascertain a particular function or metabolic lifestyle with greater confidence.

Several of our MAGs were only distantly related to the genomes of type strains. A MAG classified as Myxococcota (ANT-56) by GTDB-Tk clustered with Proteobacterial MAGs on the phylogenomic tree (Figure 8B), which might be due to its novel taxonomy. There might also be two other reasons for this: (i) lower completeness of the Myxococcota genome (75.54% completeness) and/or (ii) close relatedness of Myxococcota genome with Proteobacterial genomes, which can be supported by the recent classification of Myxococcota as a new separate phylum, which was earlier assigned to class Deltaproteobacteria (Murphy et al., 2021). Only 40 MAGs had ANI values higher than 0.95 when compared to the genomes from the GTDB reference database. Based on our current analyses, we found that only 70, 30, and 5 MAGs had a formal taxonomic nomenclature at the family, genus, and species levels, respectively. We were unable to determine the metabolic lifestyle of the MAG (ANT-120, affiliated to Alphaproteobactria) having the lowest RED value (0.66). Similarly, there were 29 other MAGs for which we were unable to determine the metabolic lifestyle based on the criteria we used in this study.

This study used high-throughput metagenomics to understand the microbial role in the marine ecosystem of the wAP. A streamlined metagenomic sequence analysis pipeline (iMAGine) was developed to process data and reconstruct MAGs. Our pipeline enabled a coverage-based approach to understand how genes were partitioned by depth and region. With this approach, we identified diverse groups of microorganisms contributing to the carbon, sulfur, and nitrogen cycle along the coastal wAP. Distinct microbial metabolisms were observed across different depth horizons. In particular, higher abundances of mixotrophic MAGs compared to heterotrophic and autotrophic MAGs were found in the shallower waters, suggesting that the dynamic pelagic environment of the coastal wAP has selected microbial populations which can adapt to rapidly changing nutrient availability. Metabolic profiles of the MAGs were phylum specific, indicating a strong link between functional guilds and taxonomy. Our results highlight the novel genetic and metabolic diversity present within Antarctic marine ecosystems and the need for future studies based on cultivable microbes to better understand the distribution of phenotypic and genotypic traits.

## Data availability statement

The datasets presented in this study can be found in online repositories. The names of the repository/repositories and accession number(s) can be found in the article/Supplementary material.

## Author contributions

AD and JB designed the study and developed the first draft of the manuscript. AD developed the iMAGine pipeline and conducted the analysis with assistance from JB. EC, RT, NE, and SD collected the samples and edited the manuscript. EC conducted DNA extractions and processed samples for sequencing. HD, DS, and OS contributed to the study design and writing. All authors contributed to the article and approved the submitted version.

## Funding

This work was funded by NSF-OPP 1846837 to JB, NSF-OPP 1440435 to HD, and NSF-OPP 2023425 to OS. JB was partly supported by the Simons Foundation Early Career Marine Microbial Ecology and Evolution fellowship. NE was funded through a SENESCYT graduate fellowship. This publication includes data generated at the UC San Diego IGM Genomics Center utilizing an Illumina NovaSeq 6000 that was purchased with funding from a National Institutes of Health SIG grant (#S10 OD026929).

## Conflict of interest

The authors declare that the research was conducted in the absence of any commercial or financial relationships that could be construed as a potential conflict of interest.

## Publisher’s note

All claims expressed in this article are solely those of the authors and do not necessarily represent those of their affiliated organizations, or those of the publisher, the editors and the reviewers. Any product that may be evaluated in this article, or claim that may be made by its manufacturer, is not guaranteed or endorsed by the publisher.

## Footnotes

^1^ https://github.com/avishekdutta14/iMAGine

^2^ https://github.com/avishekdutta14/iMAGine/blob/main/Utilities/mag_abund.py

^3^ https://bioinformatics.psb.ugent.be/webtools/Venn/

## Supplementary material

### SUPPLEMENTARY TABLE S1

Sample metadata for 48 samples.

### SUPPLEMENTARY TABLE S2

Genes considered for whole metagenome analysis.

### SUPPLEMENTARY TABLE S3

Detailed genome statistics of 137 MAGs.

### SUPPLEMENTARY TABLE S4

Detailed taxonomic classification-based genome summary.

### SUPPLEMENTARY TABLE S5

Metabolic profiles of 137 MAGs.
