# Supplementary material for: Depth drives the distribution of microbial ecological functions in the coastal western Antarctic Peninsula
Source: Front Microbiol. 2023 May 18;14:1168507. doi: 10.3389/fmicb.2023.1168507 (PMC10232865; doi:10.3389/fmicb.2023.1168507)
Supplement: Supplementary file 1 [file Data_Sheet_1.docx]

**Supplementary Figures**


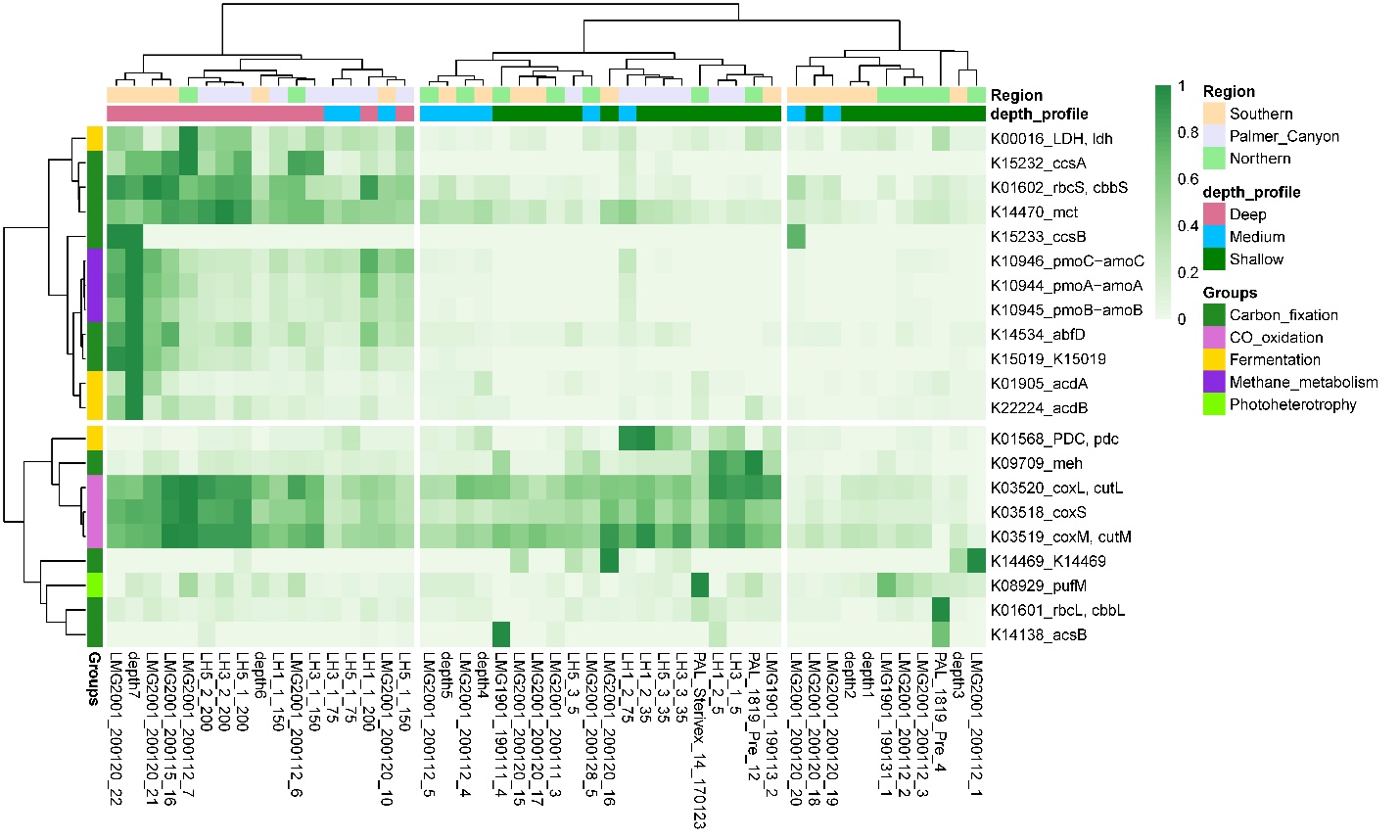


**Figure S1:** Heatmap showing normalized coverage (min-max scaled) of genes related to the carbon cycle across 48 samples. Sample clustering was done based on Bray–Curtis dissimilarity, whereas gene clustering was based on correlation.


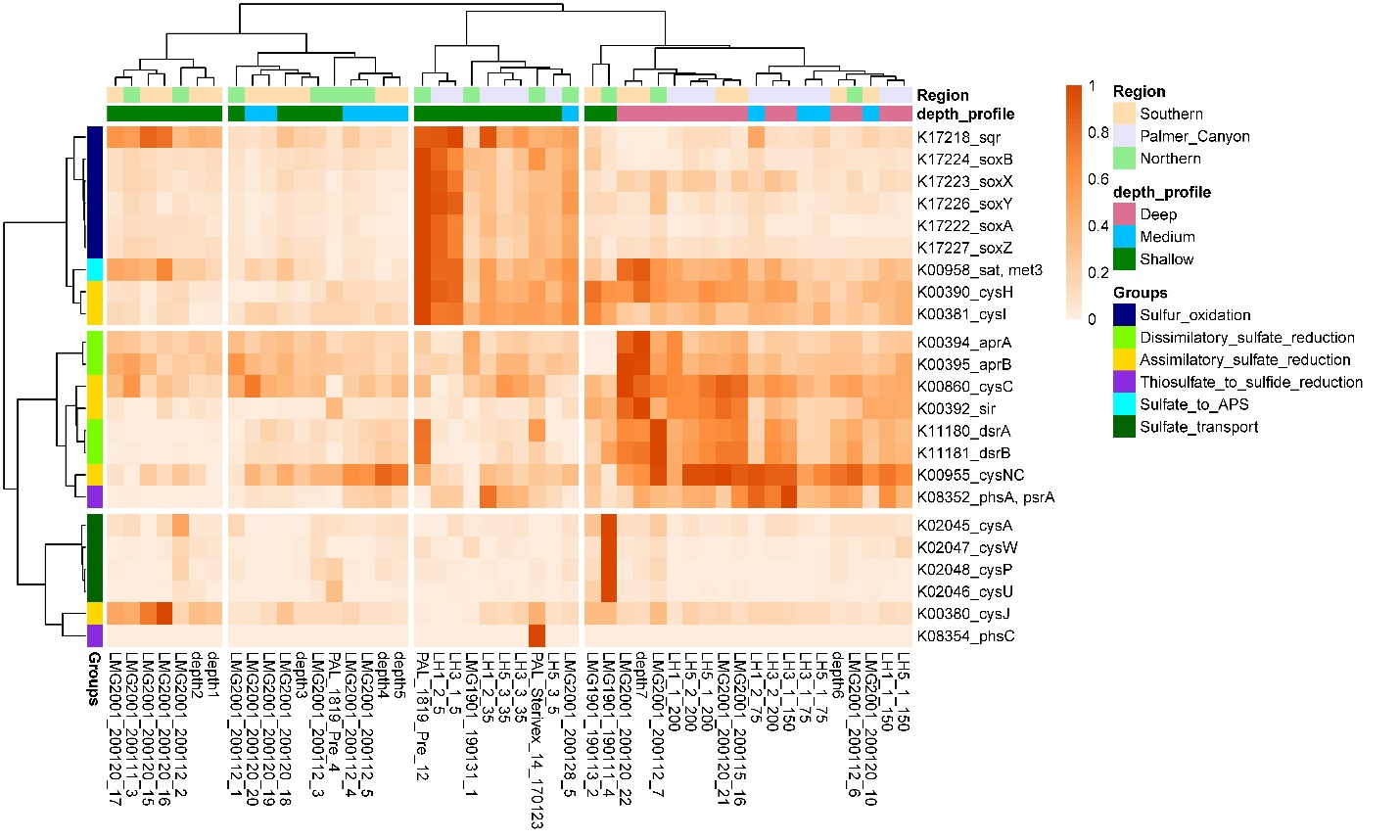


**Figure S2:** Heatmap showing normalized coverage (min-max scaled) of genes related to the sulfur cycle across 48 samples. Sample clustering was done based on Bray–Curtis dissimilarity, whereas gene clustering was based on correlation.


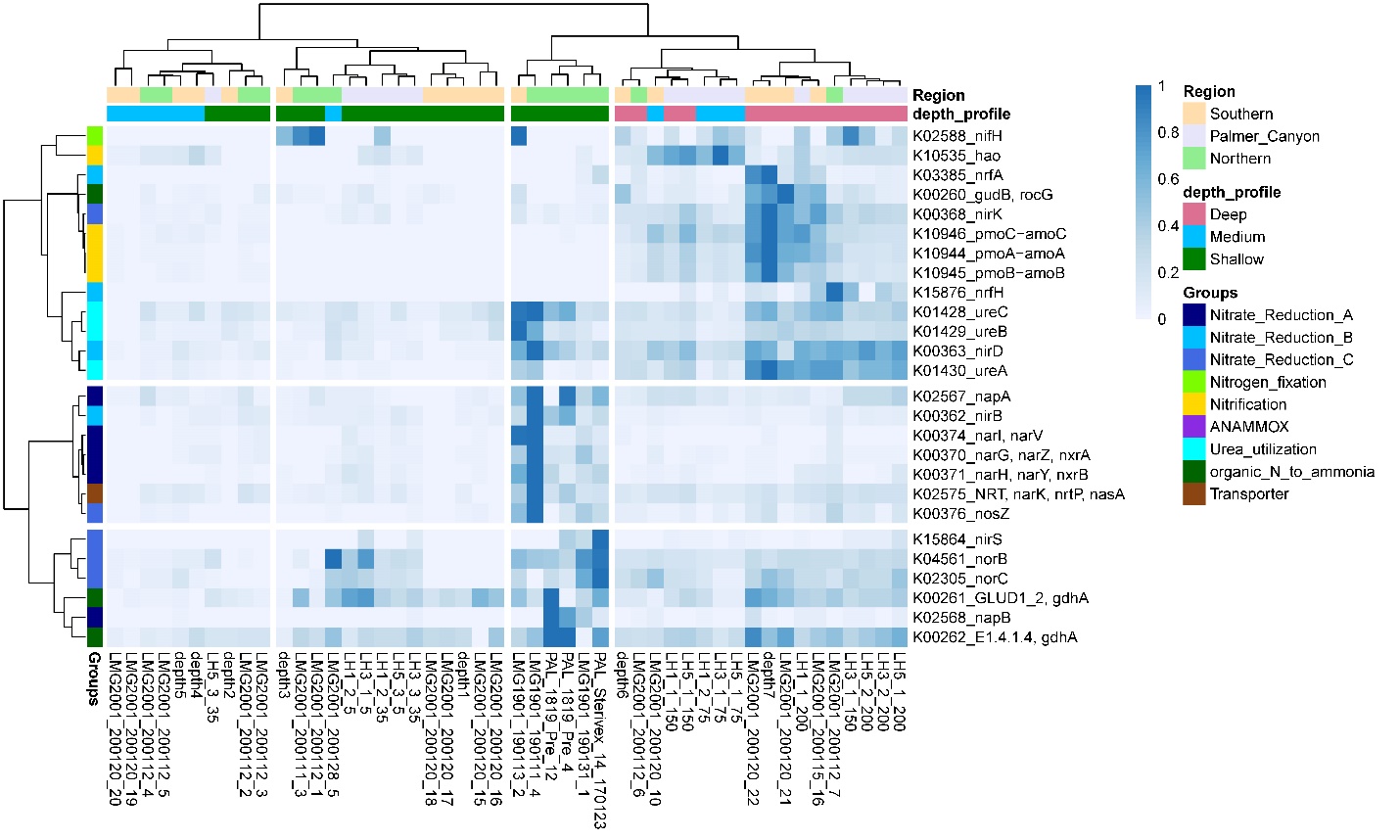


**Figure S3:** Heatmap showing normalized coverage (min-max scaled) of genes related to the nitrogen cycle across 48 samples. Sample clustering was done based on Bray–Curtis dissimilarity, whereas gene clustering was based on correlation.


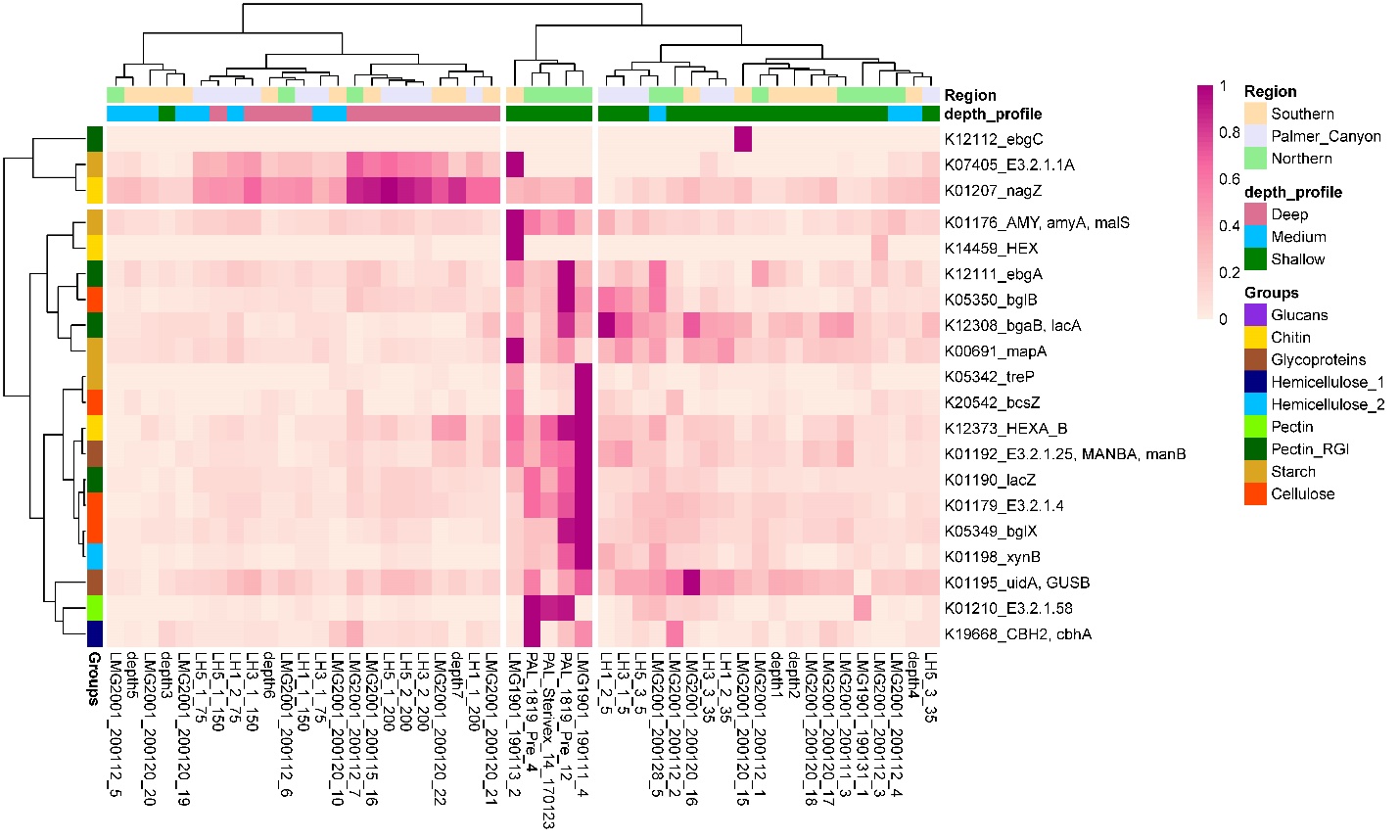


**Figure S4:** Heatmap showing normalized coverage (min-max scaled) of genes related to carbohydrate transformations across 48 samples. Sample clustering was done based on Bray–Curtis dissimilarity, whereas gene clustering was based on correlation.


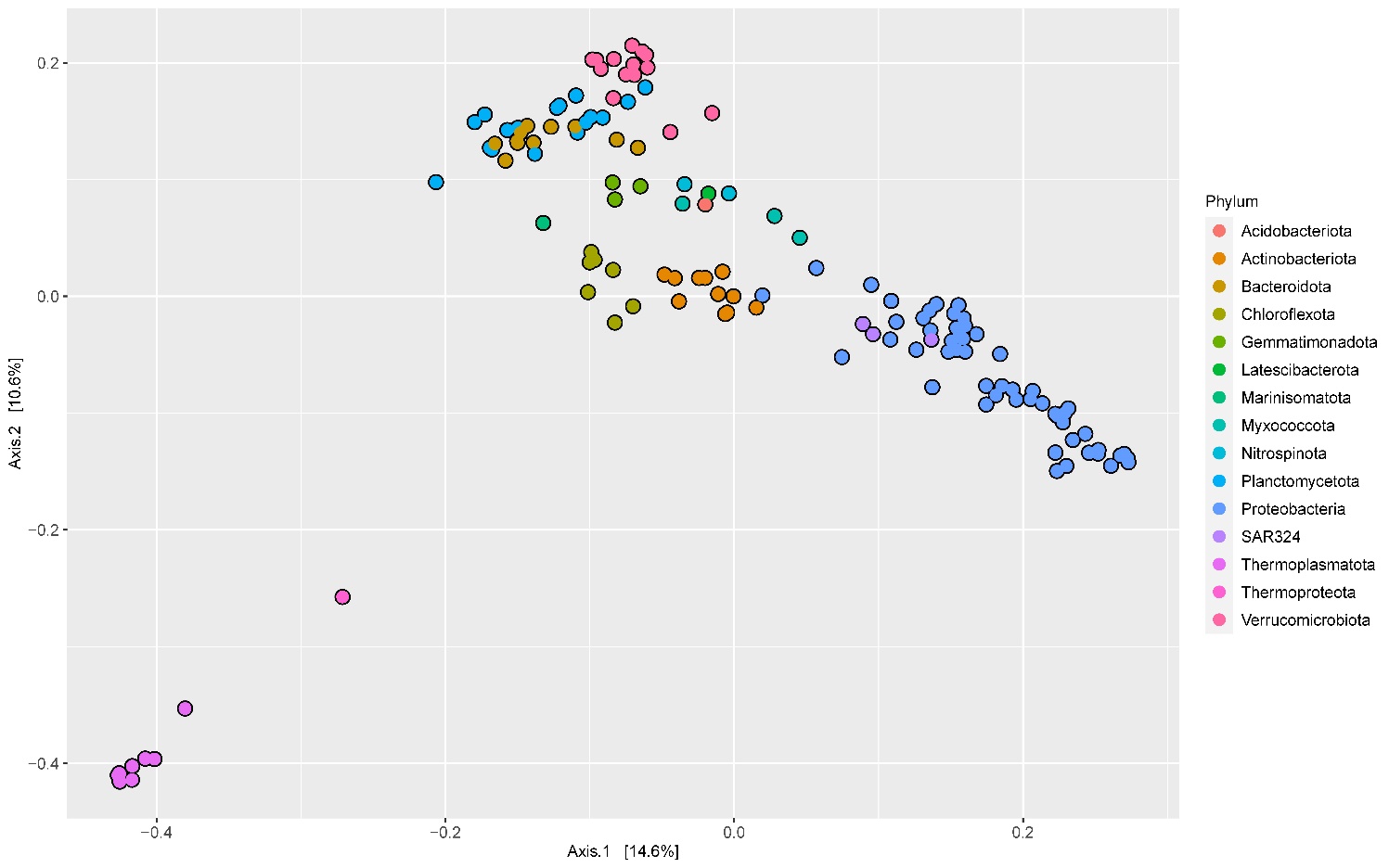


**Figure S5:** Principal Coordinates Analysis (PCoA) of binary matrix based on the presence or absence of genes predicted by Ghost Koala across 137 dereplicated bacterial MAGs.

**Figure S6:** Heatmap showing the metabolic functions in different MAGs. All the carbohydrates mentioned represent the presence of enzymes capable of degrading them. Xylan and/or xyloglucan degrading enzymes are represented by Hemicellulose in this figure. For Depth group: S represents MAGs only found in the shallower environments; M represents MAGs only found in the medium-depth environments; D represents MAGs only found in the deeper environments; MS represents MAGs found in the shallow and medium-depth environments; MD represents MAGs found in the deeper and medium-depth environments; SD represents MAGs found in the shallow and deep environments; MSD represents MAGs found in the shallow, medium, and deep environments. For Region group: N represents MAGs only found in the Northern region; PC represents MAGs only found in the Palmer Canyon region; So represents MAGs only found in the Southern environments; N_PC represents MAGs found in the Palmer Canyon region and Northern region; So_N represents MAGs found in the Southern and Northern regions; So_PC represents MAGs found in the Palmer Canyon and Southern region; So_N_PC represents MAGs found in the Palmer Canyon, Northern, and Southern region.
